# Performance Evaluation for Clinical Stroke Rehabilitation via an Automatic Mobile Gait Trainer

**DOI:** 10.3390/s23156793

**Published:** 2023-07-29

**Authors:** Chih-Jen Shih, You-Chi Li, Wei Yuan, Szu-Fu Chen, Ang-Chieh Lin, Tzu-Tung Lin, Fu-Cheng Wang

**Affiliations:** 1 Department of Mechanical Engineering, National Taiwan University, Taipei 106, Taiwan; r09522828@ntu.edu.tw (C.-J.S.); r07522829@ntu.edu.tw (Y.-C.L.); r06522835@ntu.edu.tw (W.Y.); 2 Department of Physical Medicine and Rehabilitation, Cheng Hsin General Hospital, Taipei 112, Taiwan; ch5515@chgh.org.tw (S.-F.C.); b00401112@ntu.edu.tw (A.-C.L.); b101100094@tmu.edu.tw (T.-T.L.); 3 Department of Physiology and Biophysics, National Defense Medical Center, Taipei 114, Taiwan

**Keywords:** neuro-developmental treatment, gait, rehabilitation, stroke, motor, control, pelvic

## Abstract

This paper investigates the clinical efficacy of an automatic mobile trainer for gait training in stroke patients. Neuro-Developmental Treatment (NDT) is a rehabilitation method for stroke patients that enhances motor learning through repeated practice. Despite the proven effectiveness of therapist-assisted NDT, it is labor-intensive and demands health resources. Therefore, we developed automatic trainers based on NDT principles to perform gait training. This paper modifies the mobile trainer’s intervention patterns to improve the subject’s longitudinal gait symmetry, lateral pelvic displacement symmetry, and pelvic rotation. We first invited ten healthy subjects to test the modified trainer and then recruited 26 stroke patients to undergo the same gait training. Longitudinal symmetry, lateral symmetry, and pelvic rotation were assessed before, during, and after the intervention. Most subjects show improvements in longitudinal symmetry, lateral symmetry, and pelvic rotation after using the trainer. These results confirm the trainer’s effectiveness of the modified intervention schemes in helping clinical gait rehabilitation for stroke patients.

## 1. Introduction

Stroke is the second leading cause of death worldwide [1]. The survivors usually experience physical impairments and a significant reduction in activities of daily living, especially walking capability. Many rehabilitation devices have been proposed to help stroke patients recover daily activities, e.g., walking alone. For example, West [2] developed a robotic system based on a treadmill to guide patients’ step patterns. Yano et al. [3] designed a gait rehabilitation system that steered users’ feet to simulate stair climbing or descending. Wang et al. [4] developed an active gait trainer that used six-bar linkages to guide the users’ gait patterns by controlling ankle movements. Ridler [5] designed a lightweight wearable robot to provide gait assistance and promote rehabilitation with minimal disruption for stroke patients.

The above devices guided users to follow specific movements. In contrast, Neuro-Developmental Treatment (NDT) allows patients to feel walking with minimal physical interventions [6,7]. During the NDT training, therapists guide the patients’ motions at critical moments so that the patients can intentionally drive their bodies and elicit nerve recognition to control their feet through repeated training. NDT is also known as Bobath therapy, which promotes posture control and establishes correct body mapping to improve stroke patients’ walking and motor functions [8].

NDT training is effective at improving the walking competency of stroke patients but is also time-consuming and labor-intensive for therapists. Consequently, patients might not obtain enough rehabilitation training due to the lack of therapists. Therefore, Wang et al. [9,10] designed an automatic NDT trainer to repeat therapy interventions with motors. Wang et al. [11] developed a mobile NDT trainer where the subjects could walk on the ground at self-selected speeds and receive visual feedback. Pathak et al. [12] found that adding robotic or conventional gait training to neurologic rehabilitation programs based on the Bobath (NDT) principles significantly improved balance, mobility, and quality of life in chronic stroke patients. Javier et al. [13] applied the Bobath concept to develop robot-assisted therapy for gait rehabilitation in stroke patients, gradually reducing robotic assistance and increasing patient burden.

Wang et al. [9,10] analyzed the patients’ motions and the therapist’s intervention during the conventional NDT training. They found that the therapists tended to cue the patient’s anterior-superior-iliac spine (ASIS) when the patient’s opposite heel stroked on the ground. Because NDT intervention relies on detecting the heel strike (HS), it is crucial to recognize the gait events in real time. Many researchers have studied gait patterns and recognition. For example, Wang et al. [9,10] applied a motion capture system to detect HS events. Wang et al. [11] implemented inertial measurement units (IMUs) to measure the angular velocities of the shanks [14] and developed gait detection algorithms to identify three important gait events: HS, toe-off (TO), and mid-swing (MS). Wang et al. [15] then built a long-short-term memory (LSTM) model that can recognize HS in real-time detection with an accuracy higher than 99%, even for subjects with different walking patterns.

Previous experimental results confirmed the effectiveness of the automatic NDT trainers in improving subjects’ longitudinal gait symmetry [9,10]. Two stroke patients showed improvements in the symmetries of swing phases and stride lengths after receiving training from a stationary NDT trainer [9]. In ten stroke patients receiving automatic NDT training [10], all subjects’ swing phases became more symmetric after the training. In addition to the longitudinal symmetry, pelvic locomotion also relates to walking abilities [16,17], e.g., pelvic rotation contributes to lengthening the stride [18] and energy conservation [19,20]. Hence, Wang et al. [21] analyzed therapists’ NDT interventions and concluded that the therapists tended to increase the cueing forces when observing limited pelvic rotation. Therefore, they modified the intervention patterns accordingly and invited ten stroke patients to test the modified NDT method. After receiving the modified training, nine subjects’ swing-phase symmetry and eight subjects’ pelvic rotation improved.

In addition to longitudinal gait symmetry and pelvic rotation, lateral pelvic displacement plays a role in hemiplegic gaits. The stroke patient’s body center of gravity (COG) usually biases toward the non-paretic side because of insufficient muscle strength on the paretic side. For example, the patients’ COG often biases to the right if they have left hemiplegia, and vice versa. Stroke patients walk in an asymmetrical pattern with more extensive lateral pelvic excursions than healthy people. Previous work demonstrated that increased lateral pelvic movements were associated with increased whole-body angular momentum on the frontal plane, an indicator of dynamic balance [22]. Bujanda et al. reported that impairments of the paretic limb or locomotor capacities were associated with lateral displacement of the pelvis and asymmetries for lateral accelerations among chronic stroke survivors [23]. Yang et al. [24] found that loaded walking with a rigid backpack significantly increased the mechanical energy of the stance leg and decreased lateral stability. These gait deviations may increase energy consumption and result in irregular walking patterns. Previous research also showed that lateral stabilization of the pelvis could reduce subjects’ step width [25]. MacKinnon et al. further identified that the hip abductor and adductor muscles play a crucial role in frontal plane balance during gait compared to other lower limb muscles [26]. Thus, stroke patients must receive lateral pelvic stabilization during gait training, decreasing whole-body angular momentum and improving gait stability.

We analyzed the therapists’ intervention during the traditional NDT training and found that they tended to delay the intervention when the subject’s lateral pelvic displacement (LPD) was asymmetric. Hence, we modified the NDT intervention for lateral pelvic displacement to improve longitudinal symmetry, lateral symmetry, and pelvic rotation simultaneously. This paper aimed to elaborate on the modified NDT intervention and validate whether it could simultaneously improve longitudinal symmetry, pelvic rotation, and lateral symmetry in stroke patients.

## 2. Methods

We conducted the study following the Declaration of Helsinki. Approval was granted by the local institutional Review Board (IRB: Cheng-Hsin General Hospital, Taipei, Taiwan; Protocol number: (870)110-16). All participants provided written informed consent before they participated in the study. We first recruited ten healthy participants to test the mobile NDT trainer. Then twenty-six adult stroke patients diagnosed using computed tomography or magnetic resonance imaging were recruited from Cheng Hsin General Hospital. The inclusion criteria were as follows: (1) age between 18 and 80 years, (2) suffered from stroke with Brunnstrom stage between stages III and V on the paretic lower extremity, and (3) able to walk and follow commands independently.

**Figure 1:**
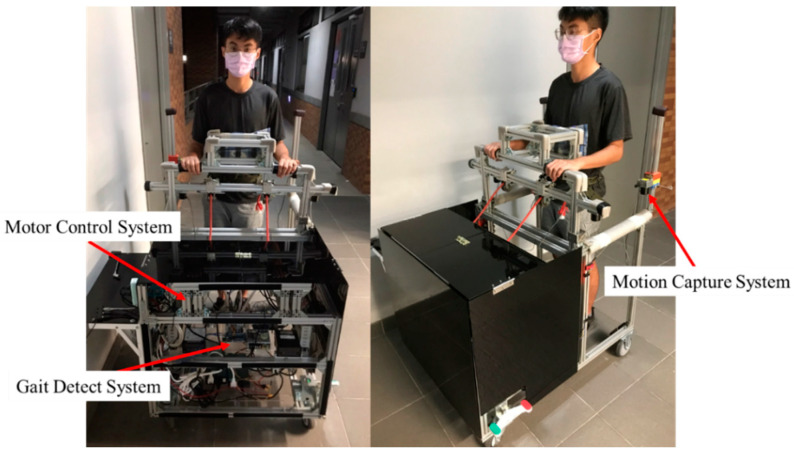
The mobile NDT trainer.

The mobile NDT trainer is shown in Figure 1, which consists of a gait detection system, a motion capture system, and a motor control system. The gait detection system applies two IMUs [27] to detect the HS events [15]. It then sends the triggering signals to the motion capture and motor control systems. The motion capture system applies an IMU and four encoders [28] to measure the user’s pelvic rotation and LPD to modify the intervention patterns. The motors [29] repeat the NDT training based on the modified intervention pattern. We invited several stroke patients and asked therapists to conduct traditional NDT training by hand. We then recorded the patients’ motions by IMUs and the therapists’ intervention by load cells [30,31,32] to derive the intervention rules. Finally, we implemented these rules for the trainer to automatically repeat the NDT intervention through the motor control system so that the trainer can be operated without the therapists’ intervention. A therapist was present during the experiments to ensure safety. Table 1 illustrates the system specifications.

**Figure 2:**
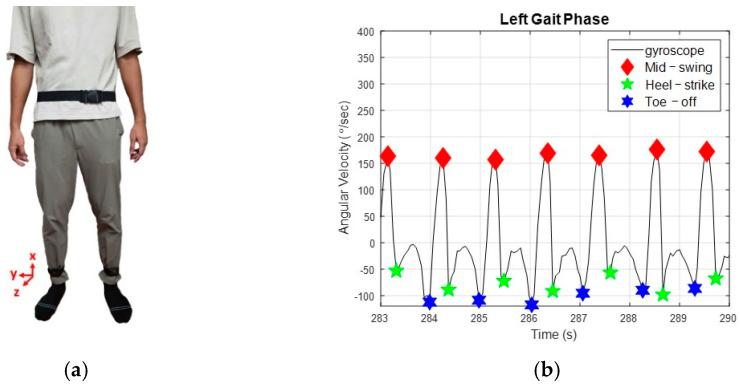
The gait patterns. (**a**) IMU attachment. (**b**) Gait responses of H8. The gait detection system can successfully identify the gait events and send triggering signals to activate the NDT intervention.

Gait cycles are generally periodic and consist of three essential gait events: MS, HS, and TO. We invited ten healthy subjects, who wore a rehabilitation gaiter on one knee to limit joint movements, to test the system; these subjects’ data are illustrated in Appendix A. We attached two IMUs to the subjects’ low limbs, as shown in Figure 2a, to measure their kinematic information during walking. Figure 2b shows the angular velocity of subject H8, where the gait detection system can successfully identify the gait events and send triggering signals to activate the NDT intervention.

We measure the subjects’ swing phase, LPD, and pelvic rotation.

**Figure 3:**
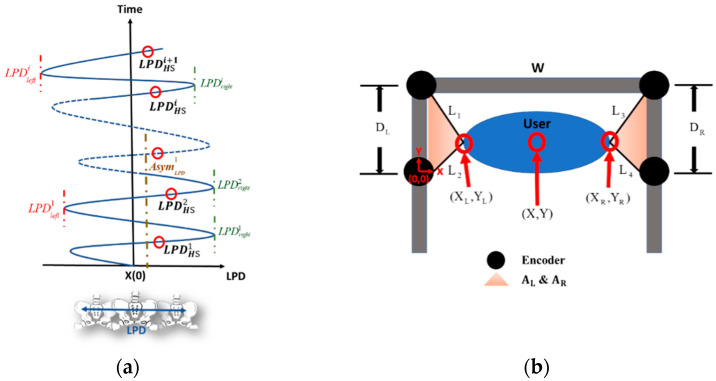

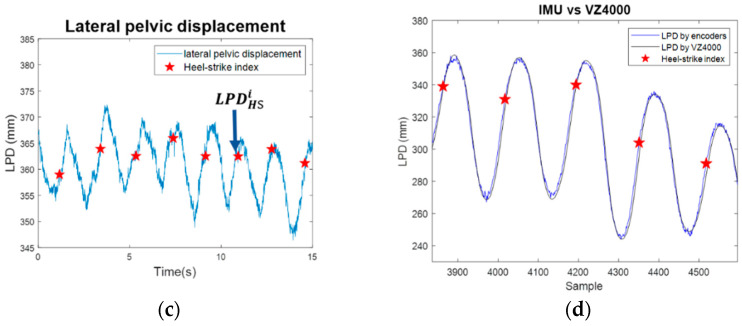
LPD measurements. (**a**) The lateral pelvic position at the *i*-th HS, marked as $LPD_{HS}^{i}$ (**b**) LPD estimation by encoders. (**c**) LPD responses of H8. (**d**) Comparison of LPD measured by the IMU and VZ4000.

**Figure 4:**
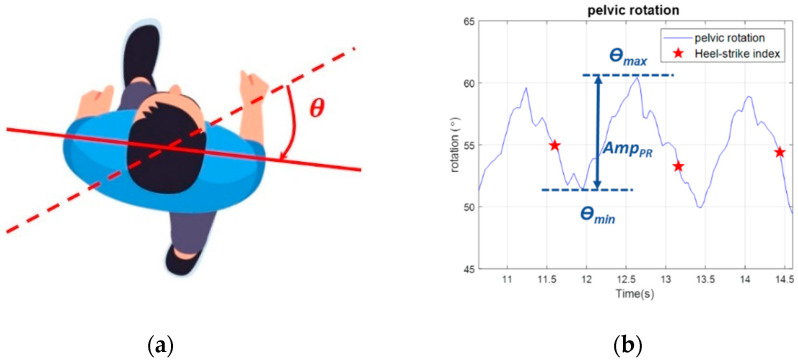
Measurement of the pelvic rotation. (**a**) Definition. (**b**) Pelvic rotation of H8.

(1) Swing phases: The swing phase of a leg is the duration between the TO and the following HS of the leg. Because human gaits are regular and periodic, we measure each subject’s gait responses and divide them into gait cycles by the HS events. For example, the gait cycles in Figure 2b were 1.82~1.85 s, while the swing phases were 0.60~0.65 s, which was about 35% of a complete gait cycle.
(2) Lateral pelvic displacement: Figure 3a shows the standard lateral position of the pelvis during walking. $LPD_{HS}^{i}$ is the lateral pelvic position with the *i*-th HS event on the hemiplegia side. $LPD_{right}^{i}$ and $LPD_{left}^{i}$ represent the lateral pelvic locations in the right-most and left-most positions during the *i*-th gait cycle (from $LPD_{HS}^{i}$ to $LPD_{HS}^{i+1}$). Because the user’s LPD locations cannot be directly measured, we applied four encoders to calculate the user’s LPD in real-time, as shown in Figure 3b. First, we apply Heron’s formula to calculate the triangle areas as follows:
  (1) $$A_{L}=\sqrt{S_{L}(S_{L}-D_{L})(S_{L}-L_{1})(S_{L}-L_{2})},\;A_{R}=\sqrt{S_{R}(S_{R}-D_{R})(S_{R}-L_{3})(S_{R}-L_{4})},$$
  in which $S_{L}=(D_{L}+L_{1}+L_{2})/2\;\mathrm{and}\;S_{R}=(D_{R}+L_{3}+L_{4})/2$. $L_{1}∼L_{4}$ are the encoder measurements. $D_{L}\;\mathrm{and}\;D_{R}$ represent the distances between encoders on the left and right, respectively. Second, we can calculate the left pelvic position $X_{L}$ and the right lateral positions $X_{R}$ using the following equations:
  (2) $$X_{L}=2\frac{A_{L}}{D_{L}},\;X_{R}=W-2\frac{A_{R}}{D_{R}}.$$
  Last, the subject’s LPD is defined as follows:
  (3) $$LPD=\frac{X_{L}+X_{R}}{2}.$$
  For example, Figure 3c shows the LPD responses of H8. We also compared the LPD measured by the encoder with that measured by an optical system, the VZ 4000 [33]. The results are shown in Figure 3d, where the two measurement systems provide similar results with an average error of 2.65 mm and a maximum absolute error of 6.65 mm. Because the errors were negligible, we applied the encoder system to evaluate the subjects’ LPD in the following experiments.
(3) Pelvic rotation: The pelvis conserves energy during gaits and lets us walk comfortably. We measured the subjects’ pelvic rotation, as shown in Figure 4a, during the NDT training with an IMU attached to the subject’s waist. The pelvic rotation $Amp_{PR}$ is defined as the maximum rotation angle between two consecutive HS events, as shown in Figure 4b. The equation is as follows:
  (4) $$Amp_{PR}=\theta_{\max}^{}-\theta_{\min}^{}$$
  where $\theta_{\max}^{}$ is the maximum pelvic angle and $\theta_{\min}^{}$ is the minimum pelvic angle in a gait cycle. For example, Figure 4b shows the pelvic rotation of subject H8 with $Amp_{PR}\approx 10^{o}$

### 2.1. Modified NDT Intervention

We developed a modified NDT intervention method that could improve the subject’s longitudinal symmetry, lateral symmetry, and pelvic rotation.

(1) Longitudinal symmetry: The motor control system repeats the NDT intervention. The analyses of the therapists’ intervention during traditional NDT training are shown in Appendix B, where the therapists tended to cue the subject’s ASIS when they observed the subject’s HS on the opposite side. Based on the analyses, we set the motors to track the following force command:
  (5) $$F(t)=\frac{\left({\bar{F}}_{\max}-{\bar{F}}_{\min}\right)}{2}\times \sin(2\pi ft)+\frac{\left({\bar{F}}_{\max}+{\bar{F}}_{\min}\right)}{2}$$
  when receiving the triggering signals from the gait detection system. ${\bar{F}}_{\max}$ is the maximum force, ${\bar{F}}_{\min}$ is the minimum force, and *f* is the frequency. In this paper, we set ${\bar{F}}_{\max}=6\;\mathrm{lb}$, ${\bar{F}}_{\min}=1\;\mathrm{lb}$, and *f* = 1 Hz based on the previous results [9,10,11,21] to simplify the experimental procedures. The frequency *f* represents the cueing patterns, which might vary among therapists. Some therapists would cue the subjects more rapidly by applying a higher-frequency command, e.g., *f* = 2 Hz, while others might cue the subjects more smoothly by applying a lower-frequency command, e.g., *f* = 0.5 Hz. Based on our experience, we set *f* = 1 to simplify the system structure. We evaluate the subjects’ longitudinal symmetry by the asymmetry of the swing phase, defined as follows:
  (6) $$Asym_{SP}\left(\%\right)=\frac{SP_{paretic}-SP_{non-paretic}}{SP_{paretic}}\times 100\%$$
  where $SP_{paretic}$ is the percentage of the swing phase on the paretic side, and $SP_{non-paretic}$ is the percentage on the non-paretic side. Stroke patients usually have a shorter duration of swing on the non-paretic side because of hemiparesis; hence, we regard the rehabilitation as effective if $\left|Asym_{SP}\right|$ becomes smaller after the training.
(2) Lateral symmetry: Stroke patients usually have a biased body COG toward the non-paretic side because of weak muscle strength on the paretic side. For example, if the patient is left hemiplegic, the COG is biased to the right, and vice versa. The asymmetry of LPD at the *i*-th gait cycle is defined as follows:
  (7) $$Asym_{LPD}=\frac{LPD_{left}^{i}+LPD_{right}^{i}}{2}+(LPD_{HS}^{i}-LPD_{HS}^{1})$$
  in which the term $\left(LPD_{HS}^{i}-LPD_{HS}^{1}\right)$ adjusts the biased LPD in different gait cycles. In general, healthy persons have symmetric LPD. If the patient is left hemiplegic, $Asym_{LPD}$ tends to be positive, and vice versa. Therefore, we regard rehabilitation as effective if $\left|Asym_{LPD}\right|$ becomes smaller after the training. The analyses of therapists’ interventions to improve LPD symmetry are illustrated in Appendix C, where the therapists tended to delay the intervention force when they observed asymmetric LPD. For simplification, we set the delay time as 0.1s when $\left|Asym_{LPD}\right|$ exceeds a threshold of 15 mm.
(3) Pelvic rotation: Stroke patients usually have limited pelvic rotation because of weakened muscles on the paretic side. Therefore, we regard rehabilitation as effective if the subject’s pelvic rotation increases. The analyses of therapists’ intervention patterns to improve pelvic rotation are shown in Appendix D, where the therapists tended to increase the applied force in Equation (5) when observing limited pelvic rotation. For simplification, we increase the maximum force by 50% when pelvic rotation is less than a threshold of 12°.

We integrated the intervention patterns mentioned above and let the motors track the following force command:

(8) $$F(t)=\frac{\left(M{\bar{F}}_{\max}-{\bar{F}}_{\min}\right)}{2}\times \sin(2\pi f(t-\delta ))+\frac{\left(M{\bar{F}}_{\max}+{\bar{F}}_{\min}\right)}{2}$$

and

$$\delta =\left\{\begin{array}{ll} 0.1, & \mathrm{if}\left|Asym_{LPD}\right|\geq 15\mathrm{mm}\\ 0, & \mathrm{otherwise} \end{array}\right.,\;M=\left\{\begin{array}{ll} 1.5, & \mathrm{if}Amp_{PR}\leq 12^{{}^{\circ}}\\ 1, & \mathrm{otherwise} \end{array}\right.$$

**Figure 5:**
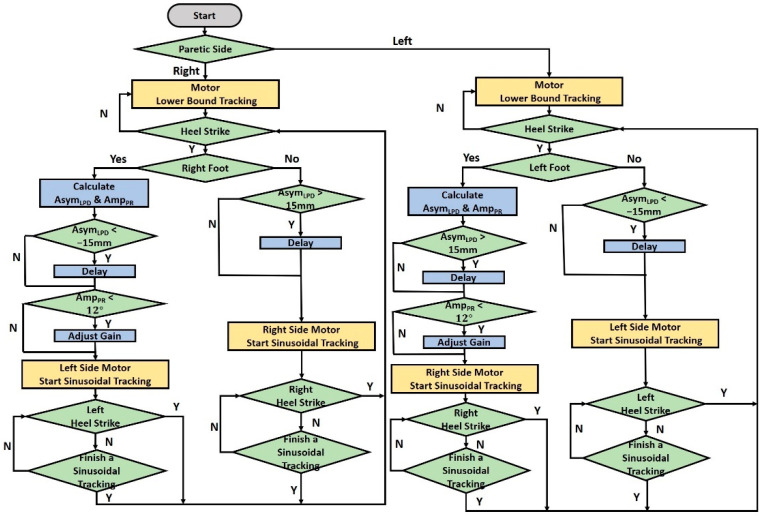
Integrated motor control processes.

Figure 5 shows the motor control procedures that integrate these three intervention patterns. First, the gait detection system measured the gait responses by IMUs and applied the LSTM model [15] to detect the HS events in real time. It sent the triggering signals to the motion capture and motor control systems. Second, the motion capture system measured the LPD and pelvic rotation. Third, the motor control system tracked a sinusoidal command after receiving the triggering signals. The right-motor commands were delayed if $Asym_{LPD}$ exceeded a threshold of 15 mm, while the left-motor commands were delayed if $Asym_{LPD}$ was less than a threshold of −15 mm. We estimated the pelvic rotation between the HS events on the hemiplegia side. The motor command on the sound side was increased by 50% if the pelvic rotation $Amp_{PR}$ was less than a threshold of 12°. Note that the maximum applied forces were 9 lb, which cued the subjects to step forward at the key gait events and would not harm the subjects. The motor tracked the sinusoidal command in Equation (8) until it was completed or when the gait detection system sent the next triggering signal.

We set the intervention parameters as constants in Equations (5) and (8) to simplify the system structure. The applied forces were small (up to 9 lb) and would not harm the subjects.

### 2.2. Experiments

We invited ten healthy subjects to test the system first. Afterward, 26 stroke patients were recruited to undergo the intervention.

(1) Healthy subjects: The healthy subjects wore a rehabilitation gaiter on one knee to limit joint movements and imitate stroke gaits. The lower limb condition of stroke patients was mimicked by applying a knee gaiter over their knee to restrict knee flexion, which is a typical extensor synergy pattern following stroke. Each healthy subject conducted the following four sets of experiments to investigate the cross-influence of the intervention methods:
  (a) With Rule 1 only, labeled as $\bar{2}\bar{3}$.
  (b) With Rules 1 and 2, labeled as $2\bar{3}$.
  (c) With Rules 1 and 3, labeled as $\bar{2}3$.
  (d) With Rules 1, 2, and 3, labeled as 23.
  Rule 1 aims to improve the longitudinal symmetry by cueing the ASIS when detecting HS on the opposite side. Rule 2 intends to improve lateral symmetry by delaying the applied forces. Rule 3 targets improving pelvic rotation by increasing cueing forces. Each subject performed the $A-B-\bar{A}$ tests in each experiment: A was before treatment, B was during-treatment, and $\bar{A}$ represented after treatment. They walked with the trainer on a straight corridor. They first walked about 20 m without motor intervention at the A stage, then about 40 m with the modified intervention at the B stage. Finally, they walked without motor intervention about 20 m at the $\bar{A}$ stage. The four sets of experiments were conducted on different days with random orders to reduce the potential carried-out effects. Each set of experiments contained the $A-B-\bar{A}$ stages without a break. However, the subjects were free to rest at any time during the experiments.
(2) Stroke patients: This study was carried out at the hospital and approved by the Institutional Review Board of Cheng Hsin General Hospital with CHGH-IRB (870)110-16. Twenty-six stroke patients conducted the experiments; each subject walked at their most comfortable speed with the trainer along a straight line. At the A stage, the subjects walked about 44 m without motor intervention. At the B stage, the subjects walked about 88 m with the modified intervention. Finally, at the $\bar{A}$ stage, the subject walked about 44 m without motor intervention. The subjects completed the $A-B-\bar{A}$ procedures continuously but could rest if necessary during the experiments. In the previous work [9,10], we compared NDT training by therapists and by an automatic NDT trainer. The results show that the NDT trainer was more effective in improving gait performance because it could provide an objective guide without getting exhausted. Considering the patients’ endurance and walking ability, we only asked them to complete one set of experiments by the trainer with Rules 1, 2, and 3. There was no break between the $A-B-\bar{A}$ procedures, though the patients could stop at any time during the experiments.

## 3. Results

The participants’ data are illustrated in Appendix A. The healthy subjects had a mean age (±standard deviation, SD) of 27.90 ± 13.38 years. The stroke patients had a mean age (±SD) of 53.19 ± 12.68 years. Forty-two percent of the patients had an ischemic stroke. Of these patients, nine were female (34.6%) and 17 were male (65.3%). The mean duration (±SD) from stroke onset was 40.07 ± 39.36 months. The Brünnstrom recovery stages for the paretic lower limb were III (n = 13), IV (n = 9), and V (n = 4). The subjects had different heights, weights, and Brunnstrom stages. Therefore, we compared each subject’s performance before the training, during the training, and after the training.

We invited ten healthy subjects to test the system. During the tests, the subjects wore a rehabilitation gaiter to imitate stroke gaits. We recorded their gait data, as illustrated in Appendix E. The healthy subjects’ performance indexes are shown in Appendix F, where the four sets of experiments were conducted on different days to avoid potential carried-out effects. For example, in the first experiment set with only Rule 1, seven subjects’ asymmetry of swing phases improved at stages B and $\bar{A}$. But, only five subjects’ asymmetry of LPD improved at stage B, and only two subjects’ asymmetry of LPD improved at stages $\bar{A}$, because Rule 1 did not aim to improve it. Similarly, only six subjects’ pelvic rotation improved at stage B, and only four subjects’ pelvic rotation improved at stages $\bar{A}$, because Rule 1 was not designed to improve pelvic rotation.

From the results, the NDT trainer effectively improved the users’ gait performance. Furthermore, the three intervention rules had no cross effects on each other. For example, Rule 1 improved the longitudinal symmetry without affecting the lateral or rotational manners. Rule 2 improved lateral symmetry without affecting the longitudinal and rotational behaviors, while Rule 3 improved the pelvic rotation without affecting the longitudinal and lateral symmetries. Table 2 shows the ratios of improved subjects in the four sets of experiments. Because there might be over-adjustments at the B stage (during treatment), we compared the results at the $\bar{A}$ stage (after training). First, applying the modified intervention method, all ten subjects had improvements in longitudinal symmetry (see entry (2, 4)); nine subjects showed improvements in the lateral symmetry (see entry (4, 4)); eight subjects showed improvements in pelvic rotation. (see the entry (6, 4)) Second, the introduction of Rules 2 and 3 did not significantly improve longitudinal symmetry (compare entry (1, 3) with entries (1, 4), (2, 3), and (2, 4)). Third, adding Rule 2 did help improve the lateral symmetry (compare entry (3, 4) with (3, 3), and entry (4, 4) with (4, 3)). Fourth, adding Rule 3 did help improve the pelvic rotation (compare entry (6, 3) with (5, 3), and compare entry (6, 4) with (5, 4)). Last, the introduction of Rule 2 did not significantly improve in pelvic rotation (see entries (6, 3) and (6, 4)), while the introduction of Rule 3 did not significantly improve in lateral symmetry (see entries (3, 4) and (4, 4)). Conclusively, with Rules 1, 2, and 3 applied simultaneously, longitudinal symmetry, lateral symmetry, and pelvic rotation improved.

According to stroke-mimicking subjects’ results, we concurrently applied Rules 1, 2, and 3 to stroke patients. Their gait data were recorded and analyzed in Appendix G, where most subjects’ gait performance was improved in terms of longitudinal symmetry, lateral symmetry, and pelvic rotation. Table 3 shows the ratio of subjects whose performance improved at the B (during treatment) and $\bar{A}$ (after training) stages. Among the 26 stroke subjects, the modified intervention method improved 16 (equivalent to 61.5%) subjects’ longitudinal symmetry, 20 (76.9%) subjects’ lateral symmetry, and 21 (80.8%) subjects’ pelvic rotation at the B stage. At the $\bar{A}$ stage, the modified intervention method improved 25 (equivalent to 96.2%) subjects’ longitudinal symmetry, 19 (73.1%) subjects’ lateral symmetry, and 20 (76.9%) subjects’ pelvic rotation. Compared with the healthy subject, the improved ratios were slightly reduced. The patients may not have enough strength to push the trainer, so the training effects could be slightly degraded.

## 4. Discussion

In this paper, we investigated the effects of a mobile trainer employing modified NDT interventions on stroke-mimicking healthy subjects and stroke patients. An LSTM model was applied to identify HS in real time. The trainer employed three intervention methods: cueing the ASIS when detecting the subject’s HS on the opposite side, delaying the applied forces when observing asymmetric LPD, and increasing the applied forces when detecting limited pelvic rotation. Our results show that most stroke-mimicking subjects improved longitudinal symmetry, lateral symmetry, and pelvic rotation after using this gait trainer. Similarly, stroke patients show improvement in all three gait parameters, although the percentage of improved subjects was less than that of healthy subjects.

Regarding the effect of modified NDT intervention on healthy stroke-mimicking subjects, we focused on the stage $\bar{A}$ (after training) to minimize the over-adjustment effect of stage B (during training). We gradually introduced rules 1 (for longitudinal symmetry), 2 (for lateral symmetry), and 3 (for pelvic rotation) step-by-step. Rule 1 was the primary intervention that could improve longitudinal symmetry. We also found that adding Rule 2 could improve lateral symmetry without compromising longitudinal symmetry and pelvic rotation, while adding Rule 3 could improve pelvic rotation without compromising longitudinal symmetry and lateral symmetry. Regarding Rule 1, we previously analyzed the therapist’s actions and test subjects’ motions during conventional NDT training for stroke patients [9,10,11]. We found that the therapists tended to apply forces on the opposite ASIS approximately when the subjects’ heels struck the ground. As cueing the opposite ASIS when heel strike stimulates the subjects to activate leg movements, Rule 1 could improve the asymmetry of swing phases of the legs. Second, in stroke patients, insufficient hip flexion, knee flexion, and ankle dorsiflexion result in compensatory upward pelvic movement at the paretic side [34,35]. Accordingly, the pelvis tends to deviate to the non-paretic side, namely, excessive non-paretic lateral pelvic displacement. Delaying the pulling force over the non-paretic pelvis at the paretic HS prolongs the stance phase on the paretic side. That could enhance the lateral pelvis displacement toward the paretic side and consequently lead to improved symmetry of LPD by Rule 2. The pelvic rotation, which contributes to lengthening the stride [18] and energy conservation [19,20], also plays a vital role in gait training. Lastly, in the typical gait pattern, the pelvic rotation results from the internal rotation of the hip joint. In stroke patients, pelvic rotation is limited due to weakness of the hip girdle muscles. Therefore, Rule 3 could improve pelvic rotation by increasing applied forces to the pelvis [21].

However, concerning the subjects with stroke, the percentage of improved subjects was less than that of healthy subjects. One possible explanation for the different responses between healthy subjects and stroke individuals is as follows: for healthy stroke-mimicking subjects, only knee joints were constrained by rehabilitation gaiters, but their pelvis and hip joints remained unrestricted. As the hip abductor and adductor muscles play a crucial role in gait stability in the frontal plane, the movements of the pelvis and hip could be facilitated immediately after NDT intervention in this group. On the contrary, stroke individuals were paretic on the whole side of the body, and instant improvement after the intervention might not be observed due to weak hip adductor and abductor muscles. Spasticity of the lower limbs could also cause abnormal synergy patterns, such as knee extension and ankle plantarflexion [25,26]. These abnormal patterns might interfere with the coordination of pelvic rotation and displacement.

Regarding longitudinal asymmetry, more stroke patients showed improvement during the $\bar{A}$ stage compared to the B stage. Contradictorily, a slightly higher proportion of stroke individuals showed improvement in LPD and pelvic rotation during the B stage compared to the $\bar{A}$ stage. A few possibilities may lead to this result. First, the longitudinal asymmetry was calculated by the difference between the swing phases of paretic and non-paretic legs. Stroke patients may adapt better lower limb control via thigh and calf muscles instead of simply through pelvic control. Therefore, the longitudinal asymmetry improved more following the NDT intervention than during the intervention. Second, pelvic rotation improved less at the $\bar{A}$ stage because the pelvis girdle muscles might not rapidly adapt to such changes in motor control. Similarly, according to previous literature, lateral stabilization could improve gait balance and reduce LPD [25]; thus, LPD improved during the intervention. However, when lateral support is discontinued, the subjects may not be able to maintain such balance; thus, lateral symmetry might not show improvement in some stroke patients.

However, some limitations in the study warrant careful consideration. First, we recruited only a small number of stroke patients. The data may not be generalizable to gait training for all stroke patients. Second, we conducted a relatively short-term intervention. The long-term effects of the intervention need to be further studied. Third, we considered only some of the gait parameters in this study. Other gait parameters might be assessed, such as endurance, motor recovery score, walking speed, and functional outcomes. Finally, pushing the trainer might constrain the users’ movements, especially for stroke patients because of insufficient muscle strength. Therefore, we designed a power-assisted trainer that automatically keeps a constant distance from the user so that the user can walk without pushing the trainer. Further large-scale controlled trials with a longer duration of intervention and functional outcome assessment are needed to confirm the long-term effect of the modified NDT intervention.

## 5. Conclusions

This paper applies a mobile NDT trainer to clinical rehabilitation training for stroke patients. The mobile trainer allowed users to receive walking rehabilitation at their preferred speeds without site limitations. First, we developed modified intervention methods that could simultaneously improve the users’ longitudinal symmetry, lateral symmetry, and pelvic rotation. We applied two wearable IMUs to detect gait events to activate NDT intervention. We also used four encoders and one IMU to measure the users’ lateral positions and pelvic rotations to adjust the intervention patterns. Second, we implemented the modified intervention methods on the mobile trainer. We recruited ten healthy subjects who wore rehabilitation gaiters to test the trainer employing the modified interventions. The experimental results confirm the safety and effectiveness of the trainer in improving their swing-phase symmetry, LPD symmetry, and pelvic rotation. Last, we recruited 26 stroke patients to conduct clinical rehabilitation via the mobile trainer. The results show rehabilitation effects for stroke patients in three indexes, which could enhance their walking ability. In the future, we plan to improve the trainer’s functions, such as motor-assisted walking, and invite more stroke patients to conduct large-scale clinical tests.

## Figures and Tables

**Table 1:** System specifications.

IMU [27]	
size	43.7 × 39.7 × 13.7 mm
weight	<25 g (with battery)
resolution	17.5 bits
sampling rate	20 to 128 Hz
range of gyroscope	±200 g
range of accelerometer	±2000 deg/s
range of magnetometer	±8 Gauss
Encoder [28]	
input voltage	0~32 VDC
output signal	0~5 V
measured distance	500 mm
maximum speed	1 m/s
step motor driver [29]	
resolution	500~125,000 steps
max pulse rate	500 kHz
input signal	4~10 V
output signal	24 V
step motor [29]	
phase	5
operating voltage	1.75 V
operating current	2.8 A/phase
static torque	16 kgf cm
load cell [30,31,32]	
range of force	200 lb
signal output voltage	0~10 V
signal output current	2 mA
accuracy	±0.02%

**Table 2:** Ratios of improved healthy subjects in the $B\;\mathrm{and}\bar{A}$ stages.

$\bar{2}\bar{3}$	$2\bar{3}$	B Stage		$\bar{A}$ Stage	
$\bar{2}3$	23				
$Asym_{SP}$		7/10	8/10	7/10	8/10
		9/10	7/10	9/10	10/10
$Asym_{LPD}$		5/10	9/10	2/10	6/10
		3/10	8/10	4/10	9/10
$Amp_{PR}$		6/10	6/10	4/10	7/10
		9/10	7/10	8/10	8/10

**Table 3:** Ratios of improved stroke subjects in the $B\;\mathrm{and}\bar{A}$ stages.

	B Stage	$\bar{A}$ Stage
$Asym_{SP}$	16/26	25/26
$Asym_{LPD}$	20/26	19/26
$Amp_{PR}$	21/26	20/26

## Data Availability

The dataset of gaits applied in this paper is available at: http://140.112.14.7/~sic/PaperMaterial/IMU_LPD_Data.zip accessed on 28 June 2020.

## Appendix A

The studies were approved by the local institutional Review Board (IRB: Cheng-Hsin General Hospital, Taipei, Taiwan; Protocol number: (870)110-16). All participants provided written informed consent before they participated in the study.

**Table A1:** Basic data of the healthy subjects.

Subject	Gender	Age	Height (cm)	Weight (kg)
H1	Male	24	172	64
H2	Male	24	161	73
H3	Male	24	180	72
H4	Male	23	175	68
H5	Male	24	163	45
H6	Male	68	168	68
H7	Male	23	178	78
H8	Female	24	153	42
H9	Female	23	150	42
H10	Male	22	172	88

**Table A2:** Basic data of the stroke patients.

Subject	Gender	Paretic Side	Age	Height (cm)	Weight (kg)	BS
S1	Female	R	58	161	70	3
S2	Female	R	42	160	81	3
S3	Male	L	43	173	75	4
S4	Male	L	39	173	80	3
S5	Male	R	52	174	66	3
S6	Female	R	49	166	65	3
S7	Male	L	68	167	78	4
S8	Female	R	22	164	75	5
S9	Male	R	48	168	60	3
S10	Male	L	44	165	81	4
S11	Male	R	61	167	64	3
S12	Male	R	41	170	73	3
S13	Female	R	62	161	57	3
S14	Male	R	44	170	73	3
S15	Male	L	74	168	75	4
S16	Male	R	61	168	70	3
S17	Male	R	63	178	73	3
S18	Female	L	69	165	59	5
S19	Female	R	55	155	78	4
S20	Male	L	73	162	69	4
S21	Male	R	76	158	51	4
S22	Female	R	40	163	85	4
S23	Male	L	54	167	83	4
S24	Male	L	52	172	75	5
S25	Female	L	49	154	75	5
S26	Male	R	44	173	77	3

**Table A3:** Comparison of the stroke patients and healthy subjects.

Subject	Stroke	Healthy
Age	53.19 ± 12.68	27.90 ± 13.38
Gender (female/male)	9/17	2/8
Type (hemorrhagic/ischemic)	15/11	-
Affected hemisphere (L/R)	16/10	-
Mean onset time (months)	40.07 ± 39.36	-
BS (III/IV/V)	13/9/4	-
Height	166.23 ± 5.83	167.20 ± 9.70
Weight	71.85 ± 8.33	64 ± 15.09

## Appendix B

The intervention method to improve longitudinal symmetry is available at: http://140.112.14.7/~sic/PaperMaterial/Sensors_2023_Appendix B.pdf accessed on 28 June 2020

## Appendix C

The intervention method to improve LPD symmetry is available at: http://140.112.14.7/~sic/PaperMaterial/Sensors_2023_Appendix C.pdf accessed on 28 June 2020

## Appendix D

The intervention method to improve pelvic rotation is available at: http://140.112.14.7/~sic/PaperMaterial/Sensors_2023_Appendix D.pdf accessed on 28 June 2020

## Appendix E

The measured IMU and LPD data of all subjects are available at: http://140.112.14.7/~sic/PaperMaterial/IMU_LPD_Data.zip accessed on 28 June 2020

## Appendix F. The Healthy Subjects’ Performance Indexes

**Table A4:** Asymmetry of the swing phase for healthy subjects.

Subject No.	$\bar{2}\bar{3}$	$2\bar{3}$	$Asym_{SP,\;A}\;$		$Asym_{SP,\;B}$ $\left(Imp_{B},\;\%\right)$		$Asym_{SP,\;\bar{A}}$ $\left(Imp_{\bar{A}},\;\%\right)$	
	$\bar{2}3$	23						
N1			13.94	15.67	8.03 (73.6)	12.83 (22.1)	15.05 (−7.4)	14.15 (10.7)
			20.10	22.61	18.88 (6.5)	17.26 (31.0)	16.96 (18.5)	18.45 (22.5)
N2			14.59	16.38	10.93 (33.5)	15.81 (3.6)	12.28 (18.8)	16.03 (2.2)
			13.56	13.45	10.90 (24.4)	12.36 (8.8)	10.23 (32.6)	12.77 (5.3)
N3			11.79	17.66	13.22 (−10.8)	10.50 (68.2)	10.14 (16.3)	11.84 (49.2)
			11.43	14.14	19.94 (−42.7)	11.32 (24.9)	15.87 (−28.0)	13.14 (7.6)
N4			21.12	18.07	17.63 (19.8)	18.66 (−3.2)	14.69 (43.8)	17.90 (0.9)
			26.85	33.82	14.48 (85.4)	14.99(125.6)	11.28 (138.0)	11.75(187.8)
N5			28.96	29.83	30.62 (−5.4)	28.59 (4.3)	27.82 (4.1)	27.98 (6.6)
			22.84	31.91	17.99 (27.0)	30.49 (4.7)	20.55 (11.1)	21.53 (48.2)
N6			12.33	19.42	16.21 (−23.9)	19.21 (1.1)	11.04 (11.7)	16.49 (17.8)
			15.18	18.50	15.04 (0.9)	17.11 (8.1)	13.66 (11.1)	12.44 (48.7)
N7			8.94	9.59	8.85 (1.0)	9.06 (5.8)	8.52 (4.9)	8.81 (8.9)
			26.91	11.45	12.68(112.2)	12.93 (−11.4)	8.19 (228.6)	11.24 (1.9)
N8			11.03	8.76	8.18 (34.8)	7.08 (23.7)	10.04 (9.9)	6.82 (28.4)
			9.98	7.70	8.61 (15.9)	6.43 (19.8)	7.54 (32.4)	6.83 (12.7)
N9			18.33	17.22	17.45 (5.0)	17.30 (−0.5)	21.96 (−16.5)	19.44 (−11.4)
			18.85	17.74	14.56 (29.5)	20.86 (−15.0)	14.74 (27.9)	12.81 (38.5)
N10			17.49	19.23	15.23 (14.8)	18.66 (3.1)	16.93 (3.3)	20.67 (−7.0)
			21.26	9.35	12.74 (66.9)	12.28(−23.9)	10.53(101.9)	8.82 (6.0)

**Table A5:** Asymmetry of the LPD for healthy subjects.

Subject No.	$\bar{2}\bar{3}$	$2\bar{3}$	$Asym_{LPD,\;A}\;$		$Asym_{LPD,\;B}$ $\left(Imp_{B},\;\%\right)$		$Asym_{LPD,\;\bar{A}}$ $\left(Imp_{\bar{A}},\;\%\right)$	
	$\bar{2}3$	23						
N1			18.79	10.69	9.11 (106.3)	4.80 (122.7)	18.87 (−0.4)	8.79 (21.6)
			32.53	25.84	38.11 (−14.6)	29.55 (−12.6)	29.36 (10.8)	21.98 (17.6)
N2			6.29	10.34	8.95 (−29.7)	4.82 (114.5)	12.90 (−51.2)	6.44 (60.6)
			5.39	9.68	3.48 (54.9)	8.18 (18.3)	5.68 (−5.1)	5.21 (85.8)
N3			6.68	7.90	4.33 (54.3)	6.45 (22.5)	6.86 (−2.6)	17.07 (−53.7)
			8.65	33.54	6.29 (37.5)	13.52 (148.1)	9.70 (−10.8)	15.77 (112.7)
N4			12.69	28.89	16.32 (−22.2)	25.29 (14.2)	14.32 (−11.4)	25.91 (11.5)
			5.58	11.37	7.53 (−25.9)	3.54 (221.2)	17.94 (−68.9)	4.71 (141.4)
N5			21.00	10.65	8.45 (148.5)	8.84 (20.5)	18.93 (10.9)	11.70 (−9.0)
			15.72	13.86	16.08 (−2.2)	11.86 (16.9)	14.67 (7.2)	4.95 (180.0)
N6			13.42	22.31	17.85 (−24.8)	22.29 (0.1)	19.03 (−29.5)	12.25 (82.1)
			5.63	36.14	10.05 (−44.0)	23.77 (52.0)	14.87 (−62.1)	20.06 (80.2)
N7			9.45	5.31	19.85 (−52.4)	5.77 (−8.0)	15.42 (−38.7)	5.99 (−11.4)
			10.61	6.81	8.80 (20.6)	5.12 (33.0)	3.78 (180.7)	2.49 (173.5)
N8			8.93	11.76	5.03 (77.5)	9.40 (25.1)	12.09 (−26.1)	19.40 (−39.4)
			3.87	6.35	3.94 (−1.8)	9.21 (−31.1)	4.09 (−5.4)	9.63 (−34.1)
N9			7.84	16.93	5.14 (52.5)	12.95 (30.7)	6.68 (17.4)	11.34 (49.3)
			14.25	23.59	9.29 (53.4)	5.56 (324.3)	10.94 (30.3)	12.63 (86.8)
N10			7.55	16.95	16.50 (−54.2)	13.95 (21.5)	13.87 (−45.6)	8.37 (102.5)
			22.32	14.01	24.72 (−9.7)	13.16 (6.5)	40.05 (−44.3)	13.76 (1.8)

## Appendix G. Gait Performance for Stroke Patients

**Table A6:** Asymmetry of the swing phase for stroke patients.

Subject (No.)	$Asym_{SP,\;A}$	$Asym_{SP,\;B}$ $\left(Imp_{B},\;\%\right)$	$Asym_{SP,\;\bar{A}}$ $\;(Imp_{\bar{A}},\;\%)$
S1	54.99	48.04 (12.65)	39.42 (28.31)
S2	21.25	30.06 (−41.45)	20.43 (3.89)
S3	15.65	13.26 (15.25)	11.43 (26.93)
S4	38.75	35.24 (9.08)	38.16 (1.53)
S5	41.75	41.51 (0.34)	30.45 (27.08)
S6	41.22	45.85 (−11.23)	37.39 (9.30)
S7	14.86	10.90 (26.65)	12.84 (13.58)
S8	18.97	21.79 (−14.91)	13.18 (30.49)
S9	39.97	33.61 (15.91)	19.44 (51.36)
S10	20.14	16.01 (20.50)	19.63 (2.52)
S11	17.29	19.16 (−10.81)	15.89 (8.10)
S12	25.54	23.27 (8.86)	18.71 (26.73)
S13	25.59	22.24 (13.09)	14.05 (45.08)
S14	25.12	24.83 (1.14)	18.72 (25.46)
S15	16.65	18.80 (−12.91)	13.39 (19.59)
S16	33.93	40.06 (−18.07)	34.39 (−1.35)
S17	16.85	18.62 (−10.48)	15.51 (7.93)
S18	25.76	20.21 (21.57)	18.46 (28.37)
S19	11.79	10.34 (12.28)	8.89 (24.55)
S20	18.61	14.13 (24.08)	9.15 (50.82)
S21	22.44	22.34 (0.42)	17.84 (20.48)
S22	25.83	23.61 (8.61)	18.80 (27.24)
S23	27.91	31.04 (−11.24)	25.61 (8.22)
S24	15.69	16.62 (−5.94)	14.88 (5.14)
S25	8.99	14.58 (−62.18)	8.41 (6.46)
S26	34.19	26.78 (21.68)	34.08 (0.33)

**Table A7:** Asymmetry of LPD for stroke patients.

Subject (No.)	$Asym_{LPD,\;A}$	$Asym_{LPD,\;B}\;$ $\left(Imp_{B},\;\%\right)$	$Asym_{LPD,\;\bar{A}}\;$ $\left(Imp_{\bar{A}},\;\%\right)$
S1	30.41	13.38 (55.99)	28.29 (6.97)
S2	31.21	14.44 (53.73)	16.83 (46.08)
S3	63.08	27.63 (56.20)	54.26 (13.98)
S4	35.46	21.53 (39.30)	35.04 (1.18)
S5	43.92	44.61 (−1.57)	64.38 (−46.59)
S6	18.85	17.66 (6.30)	14.97 (20.57)
S7	40.82	17.32 (57.58)	21.92 (46.32)
S8	53.60	21.61 (59.64)	33.11 (38.23)
S9	11.97	16.87 (−40.99)	18.59 (−55.29)
S10	11.24	7.91 (29.69)	9.93 (11.66)
S11	65.20	17.33 (73.4)	58.69 (10.0)
S12	10.2	5.77 (43.40)	9.93 (2.62)
S13	26.77	18.49 (30.93)	41.38 (−54.56)
S14	18.67	14.78 (20.85)	11.49 (38.46)
S15	23.11	32.99 (−42.73)	46.69 (−102.02)
S16	33.39	15.36 (54.00)	14.28 (57.24)
S17	29.72	23.98 (19.30)	17.66 (40.55)
S18	16.84	13.09 (22.27)	16.63 (1.26)
S19	19.81	12.08 (39.01)	11.93 (39.76)
S20	55.33	54.84 (0.89)	50.14 (9.38)
S21	67.46	40.64 (39.75)	45.06 (33.20)
S22	10.68	12.54 (−17.33)	10.75 (−0.59)
S23	25.62	26.01 (−1.52)	46.04 (−79.68)
S24	15.54	12.10 (22.15)	12.77 (17.81)
S25	23.35	26.87 (−15.07)	32.06 (−37.30)
S26	19.13	9.98 (47.79)	16.13 (15.65)

**Table A8:** Pelvic rotation for stroke patients.

Subject (No.)	$Amp_{PR,\;A}$	$Amp_{PR,\;B}\;$ $\left(Imp_{B},\;\%\right)$	$Asym_{PR,\;\bar{A}}$ $\;(Imp_{\bar{A}},\;\%)$
S1	14.95	13.50 (−9.66)	14.05 (−5.98)
S2	13.21	16.79 (27.07)	16.97 (28.41)
S3	9.3	9.45 (1.65)	9.95 (7.01)
S4	6.28	8.53 (35.84)	8.27 (31.71)
S5	14.12	13.62 (−3.51)	14.88 (5.42)
S6	10.00	11.82 (18.25)	12.46 (24.60)
S7	4.30	5.93 (37.74)	5.62 (30.65)
S8	5.13	9.06 (76.72)	7.09 (38.17)
S9	8.82	10.94 (24.06)	10.99 (24.61)
S10	9.21	10.89 (18.34)	9.87 (7.16)
S11	8.74	11.07 (26.71)	11.84 (35.45)
S12	14.46	13.20 (−8.77)	13.57 (−6.15)
S13	14.47	15.59 (7.75)	15.19 (4.99)
S14	6.48	8.24 (27.21)	7.28 (12.38)
S15	7.68	9.31 (21.23)	8.21 (6.93)
S16	16.25	16.63 (2.36)	17.73 (9.15)
S17	11.36	13.54 (19.21)	12.08 (6.40)
S18	4.7	7.71 (64.19)	5.99 (27.54)
S19	7.73	7.49 (−3.09)	5.59 (−27.75)
S20	6.01	6.59 (9.56)	5.00 (−16.86)
S21	8.71	9.20 (5.62)	7.85 (−9.82)
S22	10.57	10.65 (0.81)	12.15 (14.94)
S23	7.67	10.51 (37.04)	7.69 (0.27)
S24	4.11	6.00 (45.86)	4.28 (3.97)
S25	6.4	9.84 (53.70)	6.87 (7.20)
S26	11.54	11.11 (−3.67)	11.05 (−4.20)
